# Anthocyanin Attenuates Doxorubicin-Induced Cardiomyotoxicity via Estrogen Receptor-α/β and Stabilizes HSF1 to Inhibit the IGF-IIR Apoptotic Pathway

**DOI:** 10.3390/ijms17091588

**Published:** 2016-09-21

**Authors:** Pei-Chen Huang, Wei-Wen Kuo, Chia-Yao Shen, Yu-Feng Chen, Yueh-Min Lin, Tsung-Jung Ho, V. Vijaya Padma, Jeng-Fan Lo, Chih-Yang Huang, Chih-Yang Huang

**Affiliations:** 1 Graduate Institute of Clinical Medical Science, China Medical University, Taichung 40402, Taiwan; d27333@mail.cmuh.org.tw; 2 Department of Obstetrics and Gynecology, China Medical University Hospital, China Medical University, Taichung 40402, Taiwan; 3 Department of Biological Science and Technology, China Medical University, Taichung 40402, Taiwan; wwkuo@mail.cmu.edu.tw; 4 Department of Nursing, Mei Ho University, Pingguang Road, Pingtung 91202, Taiwan; x00003061@email.meiho.edu.tw; 5 Section of Cardiology, Yuan Rung Hospital, Yuanlin 51045, Taiwan; ab8142002@yahoo.com.tw; 6 Department of Pathology, Changhua Christian Hospital, Changhua 500, Taiwan; 93668@cch.org.tw; 7 Jen-Teh Junior College of Medicine, Nursing and Management, Miaoli 35664, Taiwan; 8 Chinese Medicine Department, China Medical University Beigang Hospital, Taichung 40402, Taiwan; jeron888@gmail.com; 9 Department of Biotechnology, Bharathiar University, Coimbatore 641046, India; padma.vijaya@gmail.com; 10 Institute of Oral Biology, National Yang-Ming University, Taipei 11221, Taiwan; jflo@ym.edu.tw; 11 Translation Research Core, China Medical University Hospital, China Medical University, Taichung 40402, Taiwan; terp3458@yahoo.com.tw; 12 Graduate Institute of Basic Medical Science, China Medical University, Taichung 40402, Taiwan; 13 Graduate Institute of Chinese Medical Science, China Medical University, Hsueh-Shih Road, Taichung 40402, Taiwan; 14 Department of Health and Nutrition Biotechnology, Asia University, Taichung 40402, Taiwan

**Keywords:** doxorubicin, anthocyanin, IGF-IIR signaling, apoptosis, estrogen receptors, cardiomyocyte

## Abstract

Doxorubicin (Dox) is extensively used for chemotherapy in different types of cancer, but its use is limited to because of its cardiotoxicity. Our previous studies found that doxorubicin-induced insulin-like growth factor II receptor (IGF-IIR) accumulation causes cardiomyocytes apoptosis via down-regulation of HSF1 pathway. In these studies, we demonstrated a new mechanism through which anthocyanin protects cardiomyoblast cells against doxorubicin-induced injury. We found that anthocyanin decreased IGF-IIR expression via estrogen receptors and stabilized heat shock factor 1 (HSF1) to inhibit caspase 3 activation and apoptosis of cardiomyocytes. Therefore, the phytoestrogen from plants has been considered as another potential treatment for heart failure. It has been reported that the natural compound anthocyanin (ACN) has the ability to reduce the risk of cardiovascular disease (CVD). Here, we demonstrated that anthocyanin acts as a cardioprotective drug against doxorubicin-induced heart failure by attenuating cardiac apoptosis via estrogen receptors to stabilize HSF1 expression and down-regulated IGF-IIR-induced cardiomyocyte apoptosis.

## 1. Introduction

The anthracycline doxorubicin (Dox), also known as hydroxyldaunorubicin, is a potent anti-tumor agent and is widely used in different types of cancer, such as breast cancer [1], skin cancer [2], and hematological cancers [3]. This is due to its high affinity for nuclear DNA and topoisomerase II [4], allowing it to intercalate into the DNA double helix and inhibit replication [5], thus resulting in DNA damage [6]. Despite its high efficiency in chemotherapy, clinical Dox treatment causes life-threatening heart failure due to the development of cardiotoxicity, forcing the treatment to become dose-limiting [7]. Multiple mechanisms have been found to be involved in Dox cardiotoxicity, including an increase in oxidative stress [8,9], reactive oxygen species (ROS) [10,11,12], and alteration in energy signaling pathways [13], thus leading to cardiac apoptosis and heart failure.

The insulin-like growth factor II receptor (IGF-IIR) induces cardiac fibrosis during myocardial remodeling and causes pathological hypertrophy [14,15]. Furthermore, we found that angiotensin II treatment causes cardiomyocyte apoptosis via destroying heat shock transcription factor 1 (HSF1), and leading to IGF-IIR activation and triggering downstream apoptotic signaling [16]. Under physiological conditions, nuclear translocation of HSF1 suppresses IGF-IIR expression. However, Dox treatment resulted in HSF1 instability and then IGF-IIR up-regulation. These results suggested that up-manipulation of HSF1 expression is a potential therapeutic strategy for alleviating cardiomyotoxicity.

Many studies revealed that cardiovascular disease (CVD) is less common in premenopausal women than in men of the same age, suggesting vascular benefits of estrogen [17]. Moreover, in animals and isolated cells, estrogen induces multiple effects that in theory should reduce heart disease risk [18,19]. Furthermore, it is well-established that up-regulation of the estrogen receptors (ERs) by estrogen inhibits cardiotoxicity through different mechanisms, such as calcineurin/NF-AT3 and the JNK1/2-NFκB pathway [20,21]. Phytoestrogens found in plants have much less estrogenic activity than natural estrogen, yet can still bind to estrogen receptors and activate certain genes, making it a new feasible direction to reduce CVD risk with fewer side effects [22,23]. Our previous investigations showed that the cardioprotective effect of a few Chinese herbs have phytoestrogenic activity to inhibit IGF-IIR expression to protect cardiomyocyte [24,25].

The phytoestrogen anthocyanin (ACN) is a member of the flavonoid family. It is found in all tissues of plants, including leaves, roots, flowers, and fruits. Due to several physiological activities that ACN possesses, including anti-inflammatory, antioxidant, peroxidation inhibition, radical scavenging, and estrogenic activity [26,27,28], it has ability to reduce the risk of CVD. There is also research indicating that ACN can decrease the cardiomyotoxicity of Dox because of its antioxidant activities [29]; however, no further studies have investigated the pathway involved in the cardioprotective properties of ACN. Here, we identified that ACN can activate ERs to increase HSF1 stability, inhibiting IGF-IIR expression and alleviating Dox-induced cardiomyocyte apoptosis.

## 2. Results

### 2.1. Dox Stimulated IGF-IIR Apoptotic Pathway and Repressed ER Expression

**Figure 1:**
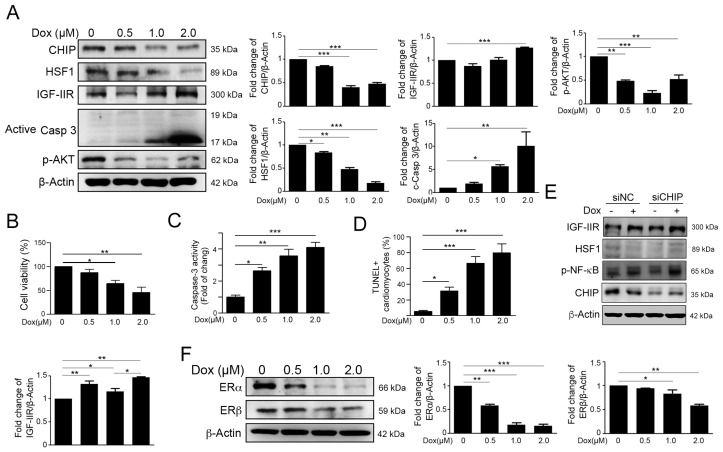
Dox stimulated the insulin-like growth factor II receptor (IGF-IIR) apoptotic pathway and repressed the expression of the estrogen receptors (ERs). (**A**) H9c2 cells are treated with different concentrations of doxorubicin for 24 h; the protein level of CHIP, HSF1, IGF-IIR, active caspase 3, and p-Akt is measured by immunoblotting. Quantification of these results is shown right (*n* = 3). * *p* < 0.05, ** *p* < 0.01 and *** *p* < 0.001; (**B**) H9c2 cells are treated with different concentrations of doxorubicin for 24 h. The cell viability was measured by MTT assay. Quantification of these results is shown (*n* = 3). * *p* < 0.05 and ** *p* < 0.01; (**C**) H9c2 cells are treated with different concentrations of doxorubicin for 24 h. The caspase-3 activities were measured by PhiPhiLux^®^-G1D2 assay. Quantification of these results is shown (*n* = 3). * *p* < 0.05, ** *p* < 0.01 and *** *p* < 0.001; (**D**) H9c2 cells are treated with different concentrations of doxorubicin for 24 h. The apoptotic cells were measured by TUNEL assay. Quantification of these results is shown (*n* = 3). * *p* < 0.05 and *** *p* < 0.001; (**E**) H9c2 cells are treated with siRNA against CHIP for 24 h, and treated with 1 µM doxorubicin for further 24 hrs. The protein level of CHIP, HSF1, IGF-IIR and p-NFκB is measured by immunoblotting; and (**F**) H9c2 cells are treated with different concentrations of doxorubicin for 24 h, the protein level of the ERs is measured by immunoblotting. Quantification of these results is shown (*n* = 3). * *p* < 0.05, ** *p* < 0.01 and *** *p* < 0.001. These data were obtained from at least three independent experiments and values represent the means ± S.D.

Previous research suggested that the cardiotoxicity of Dox acts in a dose-dependent manner. In our previous study, Dox treatment enhanced IGF-IIR accumulation via carboxyl- terminus of Hsp70 Interacting Protein (CHIP)-destabilizing HSF1 to trigger apoptosis in H9c2 cells. Therefore, we treated H9c2 cardiomyocytes with various concentration of Dox. The results of Western blotting indicated that Dox exacerbated and correlated with reductions of CHIP, HSF1, p-Akt expression, and the high expression of IGF-IIR in a dose-dependent manner (Figure 1A). Moreover, Dox triggered cardiomyocyte viability decrease (Figure 1B), increased caspase-3 activity (Figure 1C) and apoptotic cells (Figure 1D). Furthermore, CHIP deficiency led to HSF1 instability and IGF-IIR upregulation (Figure 1E).

Several lines of evidence have indicated that ERα and ERβ have the ability to protect cardiomyocyte from cardiotoxicity [30,31,32,33]. Therefore, we detected the estrogen receptor expression. After Dox treatment, we found that ERα and ERβ decreased in a dose-dependent manner (Figure 1F). These results suggested that Dox may not only induce the IGF-IIR apoptotic pathway but may also reduce ER expression following damage to H9c2 cells.

### 2.2. ACN Rescued Dox-Induced up-Rregulation of the IGF-IIR-Mediated Apoptosis Pathway

**Figure 2:**
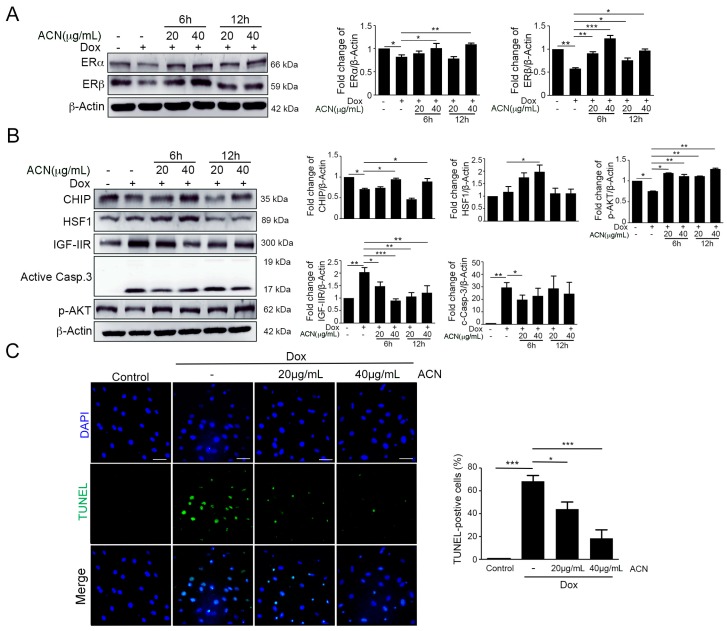
Anthocyanin enhanced ER expression and attenuated the IGF-IIR apoptotic pathway. (**A**) After H9c2 cells are treated with 1 µM doxorubicin for 6 and 12 h, they are washed with PBS, and then, fresh medium is added, followed by post-treatment with anthocyanin 20 and 40 µg/mL and incubation of cells for 24 h after doxorubicin treatment. The ERα and ERβ protein levels were measured. Quantification of these results is shown right (*n* = 3). * *p* < 0.05, ** *p* < 0.01 and *** *p* < 0.001; (**B**) after H9c2 cells are treated with 1 µM doxorubicin for 6 and 12 h, they are washed with PBS, and then, fresh medium is added, followed by post-treatment with anthocyanin 20 and 40 µg/mL and incubation of cells for 24 h after doxorubicin treatment. Proteins involved in IGF-IIR apoptotic pathway were measured by immunoblotting. Quantification of these results is shown right (*n* = 3). * *p* < 0.05, ** *p* < 0.01 and *** *p* < 0.001; and (**C**) after H9c2 cells are treated with 1 µM doxorubicin for 6 h, they are washed with PBS and fresh medium is added, followed by post-treatment with anthocyanin at 20 and 40 µg/mL, and incubation of cells for 18 h. The detection of apoptotic cells was determined by a TUNEL assay. Bars = 10 µm. Quantification of these results is shown right (*n* = 3). * *p* < 0.05 and *** *p* < 0.001. These data were obtained from at least three independent experiments and values represent the means ± S.D.

Several studies have reported that ACN is a phytoestrogen, which has estrogenic activity [34,35]. Thus, we measured protein level of ERs after treatment with ACN and Dox. We found that expression of ERs was recovered by ACN after Dox treatment (Figure 2A). To demonstrate the effects of ACN in a Dox-induced apoptosis pathway, we used Western blotting and a TUNEL (terminal deoxynucleotidyl transferase dUTP nick-end labeling) assay. In addition, results of Western blotting showed that ACN had significant protective effects on the IGF-IIR-induced apoptotic pathway (Figure 2B). Furthermore, the TUNEL assay showed that the percentage of apoptotic cells was decreased after ACN treatment (Figure 2C). These results indicated that the phytoestrogen ACN has the ability to rescue Dox-induced apoptosis via down-regulating the IGF-IIR pathway and ER expression.

### 2.3. Effects of ACN Were Attenuated by ER Antagonist ICI 182780

**Figure 3:**
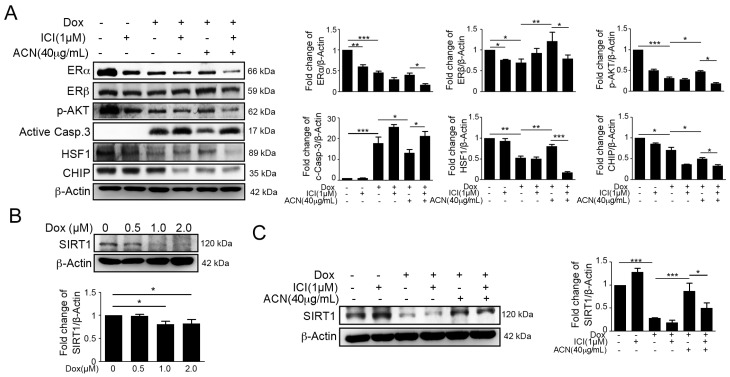
The effects of anthocyanin treatment were attenuated by the estrogen receptor antagonist ICI 182,780. (**A**) H9c2 cells were pre-treated with 1 µM ICI 182,780 for 1 h, followed by treatment with doxorubicin at 1 µM and incubation for 6 h. Then, the medium was changed to fresh medium, anthocyanin at 40 µg/mL was added, and the cells were incubated for 18 h before measurement by immunoblotting. Quantification of these results is shown right (*n* = 3). * *p* < 0.05, ** *p* < 0.01 and *** *p* < 0.001; (**B**) the level of SIRT1 protein expression after different concentrations of doxorubicin for 24 h was investigated by immunoblotting. Quantification of these results is shown (*n* = 3). * *p* < 0.05; (**C**) H9c2 cells were pre-treated with 1 µM ICI 182,780 for 1 h, followed by treatment with doxorubicin at 1 µM and incubation for 6 h. Then, the medium was changed to fresh medium, anthocyanin at 40 µg/mL was added, and the cells were incubated for 18 h before measurement by immunoblotting. Quantification of SIRT1 epxression is shown right (*n* = 3). * *p* < 0.05, ** *p* < 0.01 and *** *p* < 0.001.

To further confirm whether ERs play a role in the protective effect of ACN against the Dox-induced IGF-IIR apoptotic pathway, we used pre-treatment with the ER antagonist ICI 182780 to inhibit ER expression, followed with Dox and/or ACN treatment. The results of Western blotting revealed that ICI significantly enhanced the Dox-induced IGF-IIR apoptotic pathway (Figure 3A).

Moreover, we found that ICI remarkably ameliorated the cardioprotective effects of ACN, including up-regulation of CHIP and HSF1 expression (Figure 3). Taken together, these results indicated that ACN may protect cardiomyocytes against DOX through ER expression enhancement. Recently, it has been reported that SIRT1 is highly expressed in hearts and has the ability to protect cardiomyocytes from injury [36,37,38]. Our previous study demonstrated that ANG II degrades SIRT1, resulting in HSF1 acetylation, and induces IGF-IIR-mediated apoptosis [16]. Therefore, we examined SIRT1 expression by immunoblotting. We found that DOX treatment significant decreased SIRT1 expression, but ACN alleviated the DOX-induced SIRT1 decrease (Figure 3B,C). These results indicated that ACN may also be involved in the SIRT1-HSF1 pathway to protect cardiomyocyte.

### 2.4. Function Recovering Effect of ACN in Dox-Treated Hearts in Vivo

**Figure 4:**
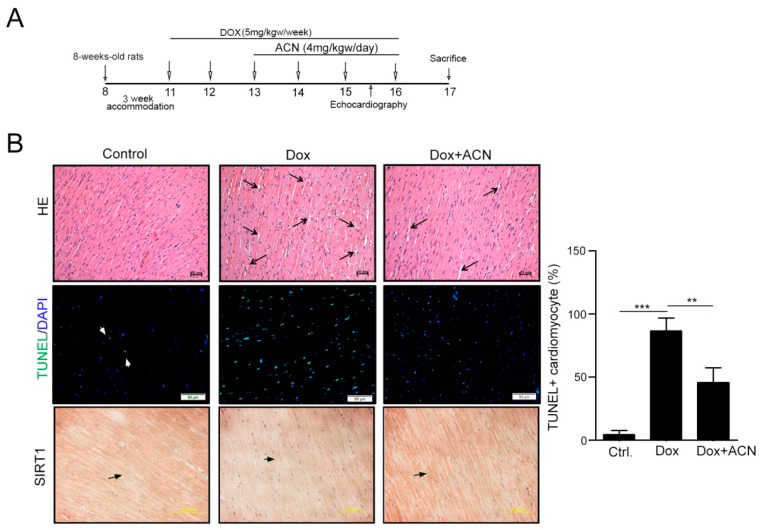
Echocardiographic assessments and histopathological analysis of rat left ventricular cells after doxorubicin and anthocyanin treatment. (**A**) The schematic procedure of DOX and HSF1A administration; (**B**) histopathologic analysis of heart tissue sections stained with H and E. Magnification: 200×; bars = 50 µm. An enlarged interstitium was observed in the doxorubicin-treated rat hearts, and the arrows indicate the myocardial interstitium. The expression of TUNEL + cardiomyocytes and SIRT1 expression were evaluated by immunohistochemistry (IHC) and TUNEL assay. Quantification of TUNEL + cardiomyocytes from each group is shown right (*n* = 3 per group). These data were obtained from at least three independent experiments and values represent the means ± S.D. ** *p* < 0.01 and *** *p* < 0.001.

We then analyzed the effect of ANC on Dox-induced cardiotoxicity in an animal model. After intraperitoneal injection of Dox for six weeks with/without oral gavage of ACN for the last four weeks, the heart functions were analyzed by echocardiography (Figure 4A). The results of echocardiography showed that the function of rat hearts was dramatically impaired by DOX. Interestingly, these functions were recovered after ACN treatment. Both ejection fraction (EF) and fractional shortening (FS) were significantly rescued. Moreover, ACN recovered the DOX-induced damages of the thicknesses of the left ventricular posterior wall (LVPW) and interventricular septum (IVS) (Table 1). The appearance of heart tissue showed that the arrangement of myocytes was disordered, and the interstitial space increased in the Dox treatment group. However, these cardiotoxicity effects were remarkably alleviated by ACN treatment, including a more ordered myocyte arrangement and less interstitial space (Figure 4B). Moreover, the TUNEL + cardiomyocyte induced by Dox also significantly reduced in Dox-ACN group (Figure 4B). Furthermore, by immunohistochemistry staining, we found that the Dox-induced SIRT1 decrease was recovered by ACN. These results suggest that ACN has the ability to recover heart function following Dox injury.

### 2.5. Effects of Rat Left Ventricular Protein Expression as a Result of Dox and ACN Treatment in Vivo

**Figure 5:**
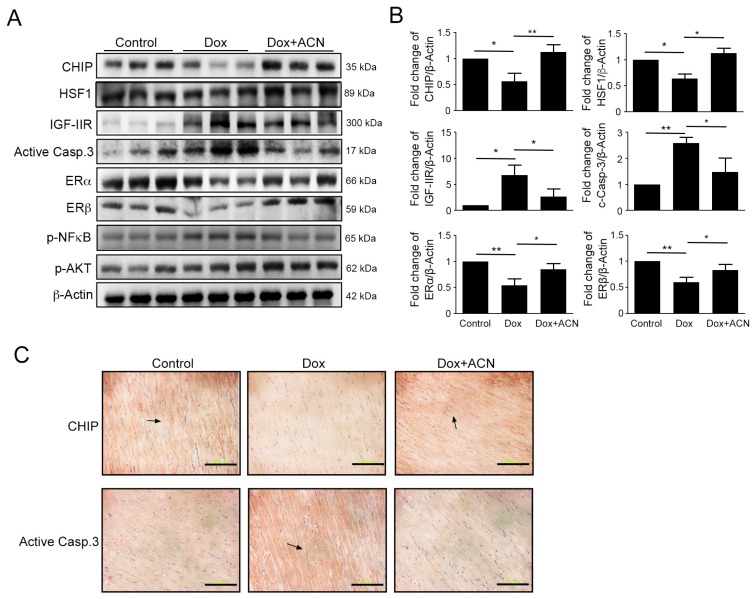
Protein expression of the left ventricles of rat hearts after six weeks of doxorubicin and anthocyanin treatment. (**A**,**B**) The left ventricles of hearts were excised and homogenized. The cell lysates were quantified and analyzed via immunoblotting. The expression of the IGF-IIR signaling pathway protein and the expression of the apoptosis marker caspase-3 and ERs were estimated via immunoblotting. Quantification of the results is shown right (*n* = 3 per group). * *p* < 0.05 and ** *p* < 0.01; and (**C**) immunohistochemical detection of CHIP, active caspase-3 expression. Arrows indicated the expression of CHIP and active caspase-3, respectively. Magnification: 400×; bars = 10 µm.

Moreover, the results of western blotting from left ventricular heart tissues showed that DOX significantly increased IGF-IIR and cleaved caspase-3 expression, accompanied by decreased expression of p-Akt, ERα, ERβ, and the upstream protein, CHIP (Figure 5A,B). The results of the IHC stain confirmed that cleaved caspase-3 and CHIP expression were increased (Figure 5C). Furthermore, SIRT1 expression also strongly recovered following ANC treatment, examined by immunohistochemistry (Figure 4B). Overall, these results showed that ACN has the ability to alleviate the Dox-induced cardiotoxicity via ER expression and IGF-IIR apoptotic pathway inhibition.

## 3. Discussion

**Figure 6:**
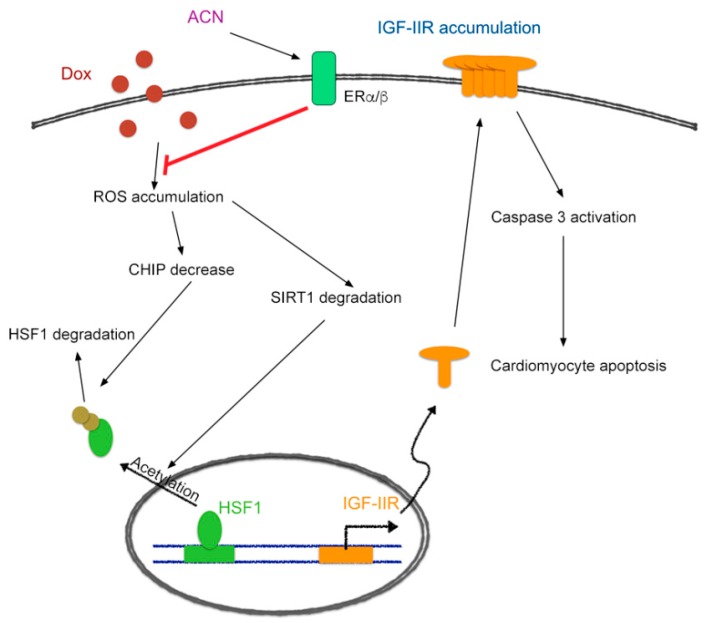
Schematic diagram of how ACN attenuates doxorubicin-induced cardiomyotoxicity through ERα/β to up-regulate CHIP-mediated HSF1 nuclear translocation and SIRT1-mediated HSF1 activation to inhibit the IGF-IIR apoptotic pathway.

In this study, we demonstrated a new mechanism through which anthocyanin protects cardiomyoblast cells against doxorubicin-induced injury. We found that anthocyanin decreased IGF-IIR expression via estrogen receptors and stabilized HSF1 to inhibit caspase-3 activation and apoptosis of cardiomyocytes (Figure 6). This may provide another new strategy for inhibition of doxorubicin-induced cardiomyotoxicity.

Doxorubicin is a highly effective anti-cancer agent. The anti-cancer function is believed to be attributed to its inhibition of topoisomerase II and DNA replication [39]. Although it has highly beneficial effects against cancer, it is also well-established that doxorubicin treatment will cause life-threating cardiovascular damage [40]. Some molecular mechanisms of cardiomyocyte apoptosis induced by doxorubicin have been reported, such as the most widely held hypotheses: production of reactive oxygen species (ROS) by the electron acceptor doxorubicin [41], mitochondrial iron accumulation [42], and intracellular calcium dysregulation [43], suggesting that apoptosis inhibition and up-stream signal blockage may potentially block the negative side effects of doxorubicin. Our previous studies revealed that IGF-IIR accumulation significantly induces not only cardiomyocyte hypertrophy but also apoptosis, and this process may be achieved through several downstream signals, including calcinurin [44,45], phosphorylation of PLCβ [46], and Gαq [47].

In addition, we recently found that the upstream signal triggers for the IGF-IIR apoptotic pathway, HSF1 and CHIP interference, are also impacted by doxorubicin, the mechanisms of ACN on cardioprotection through regulation of ERα, ERβ, and SIRT1/HSF1 signaling. Estrogen has been reported to reduce the risk of cardiovascular system via multiple effects [18,48,49] and reduce hypertrophy in ER-dependent mechanism [33]. Moreover, evidences have enlightened that estrogens and phytoestrogens shared the structural similarities and their proactive effects in the reduction of cardiovascular risk, such as genistein, calycosin, provinols, and delphinidin [31,35,50,51]. Furthermore, the cardioprotective ability of phytoestrogen anthocyanin may be attributed to its capacity to enhance NOS activity, NO release [52,53], antioxidative activity [54] and NF-κB reduction [55]. Consistent with these findings, our earlier studies also found that Chinese herb extracts, Danshen and Dungshen, exert strong estrogenic activity to activate both ERs against IGF-IIR apoptotic signaling in cardiomyocytes [24,30,56]. Here, we found that doxorubicin treatment significantly decreased levels of both ERα and ERβ, particularly in ERα. Moreover, the ERα expression level is more significantly down-regulated, compared to ERβ as Dox treatment in cell model, implying that ERα might be as a key target of doxorubicin. The black rice anthocyanin significantly inhibited Dox-induced cardiomyocyte apoptosis and improved cardiac function via ERs in vitro and in vivo. Indeed, several studies indicated that phytoestrogens exert their benefits via ERα activation, such as blackcurrant anthocyanins and red wine anthocyanins delphinidin [33,34,35]. However, we still cannot exclude out the importance of ERβ because of the dramatically drop in animal model as Dox administration. But the black rice anthocyanin clearly up-regulated ERα and ERβ expression level to improve cardiac function. In accordance with our findings, Liu et al. recently reported that calycosin, a phytoestrogen from Radix astragali, exhibits anti-oxidative stressed-induced apoptosis by activating both ERα and ERβ in cardiomyocytes [31]. We need to further investigate the role of ERβ in Dox-induced cardiomyocyte apoptosis in future. 

Our previous results have established the IGF-IIR apoptotic signaling pathway can be inhibited by found that some phytoestrogens have the ability to reduce cardiac apoptosis via inhibiting it. Here, we used the phytoestrogen anthocyanin and for the first time found that not only can it reduce cardiomyocyte apoptosis through ER up-regulation, but that it may also have the ability to inhibit IGF-IIR apoptotic pathway through its up-stream signal, the HSF1 pathway, and through ER expression.

It has been reported that generation of oxidative stress is responsible for the activation of NF-κB [57] and the Dox-induced cardiomyocytes apoptosis is due to the activation of NF-κB [58,59]. SIRT1 is a NAD+-dependent deacetylase, which has been reported to influence cellular metabolism via changing the energy in the nucleus and mitochondria [60]. It participates in biological functions related to development of heart failure, including regulation of energy production, oxidative stress, intracellular signaling, angiogenesis, autophagy, and cell death/survival [61]. Previous studies indicated that SIRT1 enhanced the expression of AMPK and improved cardiac function via estrogen-dependent mechanism [36,62]. SIRT can activate eNOS to protect the heart against oxidative stress [63] and inhibit NF-κB-related inflammation [61,64]. Therefore, Dox-induced oxidative stress and SIRT1 decrease may both contribute to NF-κB activation to promote apoptosis in cardiomyocyte.

Additionally, our recent studies indicated that SIRT1 degradation resulting in HSF1 acetylation and dysfunction, which led to angiotensin II-induced IGF-IIR hypertrophy, suggesting that SIRT1 might be the target of Dox-induced cardiotoxicity [16]. In support with our findings, Sin et al. indicated that SIRT1 played an important role in resveratrol protection against doxorubicin-induced cardiotoxicity in aged hearts and angiotensin II-induced cardiomyocyte apoptosis in hypertensive heart failure [65]. CHIP has been reported to interact with HSF1 for HSF1 protein stability and nuclear translocation [66]. Moreover, CHIP exerts its cardioprotective effect via regulating protein stability during heart failure [67,68]. In accordance with these findings, our results showed that anthocyanin up-regulated CHIP expression to stabilize HSF1, which can inhibit IGF-IIR expression. Taken together, our observations revealed the protective functions of anthocyanin may be contributed from directly up-regulating ERα/β to inhibit oxidative stresses-induced CHIP and SIRT degradation. CHIP can maintain HSF1 stability and nuclear translocation, and SIRT1 can sustain HSF1 DNA-binding activity to inhibit IGF-IIR expression.

## 4. Materials and Methods

### 4.1. Chemicals and Antibodies

Anthocyanin extracted from black rice was provided from Asia University (Taichung, Taiwan). A screening experiment for anthocyanin components in the extracts of black rice was done using HPLC with a DAD monitored spectra at a wavelength of 520 nm. All HPLC chromatograms obtained showed only two intense peaks at the retention times of 11.39 and 14.80 min. The positive ion scan mass spectra obtained by liquid chromatography-electrospray ionization-tandem mass spectrometry (LC-ESI-MS) of these peaks contained protonated molecular ions ([M + H^+^]) of *m*/*z* 449 and 463. These fragmentations were from the aglycone residues and showed themselves in the mass, therefore, identified as anthocyanin monoglucoside. To determine individual anthocyanin components in each black rice extract, the calibration curves of standard cyanidin-3-*O*-glucoside and peonidin-3-*O*-gluco side were constructed in the concentration range of 5.00–40.0 mg/mL for anthocyanins. The anthocyanins extracted from black rice was diluted in double-distilled water, and stored at −20 °C [69,70,71]; doxorubicin (for cells) was purchased from Sigma Aldrich (St. Louis, MO, USA), diluted in dimethyl sulfoxide, and stored at −20 °C; doxorubicin (for animals) was from LC Laboratories (New Boston, NH, USA); the ER antagonist ICI 182780 was purchased from TOCRIS (Bristol, UK), diluted in dimethyl sulfoxide, and stored at 4 °C.

The primary antibodies used were β-actin (sc-47778), p-Akt (sc-7985), HSF1 (sc-9144), ERα (sc-7207), SIRT1 (sc-15404) and were purchased from Santa Cruz Biotechnology (Dallas, TX, USA); ERβ (ab3576) and IGF2R (ab124767) were purchased from Abcam plc. (Cambridge, UK), and cleaved caspase-3 (#9664) was purchased from Cell Signaling Technology, Inc. (Danvers, MA, USA).

### 4.2. Cell Culture and Treatment

H9c2 cardiomyoblast cells were cultured in Dulbecco’s modified Eagle’s medium (Sigma Aldrich, St. Louis, MO, USA) with 10% cosmic calf serum (HyClone, Logan, UT, USA), 100 µg/mL streptomycin, 25 mM glucose, and 2 mM glutamine supplementation. All cells were maintained in 5% CO_2_ at 37 °C in an incubator. The experimental groups were treated with different drugs after being seeded for 24 h and grown to 80% confluence.

### 4.3. Western Blot Analysis

To obtain total protein, cells were harvested and were lysed by lysis buffer (50 mM Tris, pH 7.5, 0.5 M NaCl, 1.0 mM ethylenediamine tetraacetic acid (EDTA)), pH 7.5, 10% glycerol, 1 mM β-mercaptoethanol (BME), 1% IGEPAL-630, and a proteinase inhibitor cocktail (Roche Molecular Biochemicals, Berlin, Germany) for 30 min, followed by centrifugation at 12,000× *g* for 10 min. The collected supernatants were quantified by Bradford assays (Bio-Rad, Hercules, CA, USA). The proteins were separated by 8%–13.5% SDS-PAGE and transferred onto polyvinylidene difluoride (PVDF) membranes (Millipore, Belford, MA, USA). Nonspecific protein binding was blocked by Tris-buffered saline Tween-20 (TBS-T) containing 5% skim-milk for 1–2 h, and blotted by specific primary antibodies (1:1000 diluted in TBS-T) at 4 °C overnight. Protein signals were measured after being incubated in HRP-conjugated secondary antibodies (1:3000 diluted in TBS-T) at RT for 1 h, followed by Immobilon Western Chemiluminescent HRP substrate (Millipore, Billerica, MA, USA). The immunoblots were evaluated using an Αmager 2200 digital imaging system (Digital Imaging System, Commerce, CA, USA).

### 4.4. TdT-Mediated Digoxigenin-dUTP Nick-End Labeling (TUNEL) Assay

H9c2 cell apoptosis was detected by a TUNEL assay, which was performed with a commercial in situ apoptosis detection kit (Roche Molecular Biochemicals). The cells were cultured in eight-well Millicell EZ slides (Merck Millipore, Darmstadt, Germany), fixed with 4% paraformaldehyde (in PBS) for 30 min, and permeabilized with 0.1% Triton X-100 for 15 min. After being stained by TUNEL fluorescence reagent and DAPI (4′,6-diamidine-2-phenylindole dihydrochloride, Sigma-Aldrich) for nuclei, the cells were observed by fluorescence microscopy (Olympus, Tokyo, Japan). UV light detected all nuclei (blue) and a wavelength in the range of 515–565 nm detected TUNEL-positive nuclei (green). Three independent experiments were then averaged and statistically analyzed.

### 4.5. Cell Viability Assay

Cell viability was estimated using a colorimetric assay based on the conversion of tetrazolium dye (MTT [3-(4,5-dimethylthiazol-2-yl)-2,5-diphenyltetrazolium-bromide]) into a blue formazan product. After harvesting and washing twice with PBS, the cells were cultured in phenol red-free DMEM (1 mL) with MTT (0.5 mg/mL) at 37 °C for 4 h. The cells were then incubated in isopropanol (1 mL) with shaking for 10 min, aspirated and measured spectrophotometrically at 570 nm.

### 4.6. Flow Cytometric Analysis for Caspase-3 Activity

Following treatment, cells were washed with PBS, then treated with trypsin and harvested. Cells were incubated with PhiPhiLux^®^-G1D2 (A304R1G-5, OncoImmunin, Inc., Gaithersburg, MD, USA) at 37 °C for 30–60 min and washed by PBS before flow cytometric analysis.

### 4.7. Animal Models

Eight-week-old male Wistar Kyoto (WKY) rats (Lasco Biotechnology Co., Ltd., Taipei, Taiwan) were randomly separated into three groups: Control group (*n* = 3), Dox treatment group (*n* = 3), and Dox with ACN treatment group (*n* = 3). All rats were provided with food and water ad libitum, with a 12/12 h light/dark cycle in ambient temperature (22–24 °C), and were allowed to adapt to the environment for three weeks before the experiment. Dox treatment started when rats were 11-weeks-old (5 mg/kg weekly, intraperitoneal injection) and continued for six weeks, and ACN treatment (4 mg/kg per day, gavage feeding) started three weeks after Dox injection began, for four weeks. The protocol (Protocol number: 2016-059, 2015-12-22) was approved by the Institutional Animal Care and Use Committee of China Medical University, Taichung, Taiwan, and the principles of laboratory animal care (NIH publication) were followed.

### 4.8. Tissue Extraction

The left ventricles were separated from the rats, homogenized and the tissues were extracted. The samples were placed in lysis buffer (50 mM Tris, pH 7.5, 0.5 M NaCl, 1.0 mM ethylenediamine tetraacetic acid (EDTA), pH 7.5, 10% glycerol, 1 mM β-mercaptoethanol (BME), 1% IGEPAL-630, and a proteinase inhibitor cocktail (Roche Molecular Biochemicals) at a ratio of 200 mg/mL buffer and were centrifuged at 12,000× *g* for 10 min. The supernatant was collected and stored at −80 °C.

### 4.9. Hematoxylin-Eosin Stain

After heart tissues were excised, hearts were fixed in formalin, dehydrated by graded alcohols, and embedded in paraffin wax. The 0.2 µm-thick tissue section were deparaffinized by immersing in xylene and rehydrated, followed by staining of hematoxylin and eosin. Finally, tissues were soaked in xylene twice and dried, and morphological changes in the stained sections were examined under Zeiss Axiophot microscopy.

### 4.10. Immunohistochemistry Staining

The tissue sections were dehydrated through graded alcohols and embedded in paraffin wax. The slides were heated in Dewax and HIER Buffer H (Thermo Fisher Scientific, Inc., Waltham, MA, USA) for 20 min, and endogenous peroxidase activity with 3% H_2_O_2_ was blocked, followed by blocking with 5% BSA for 10 min. The sections were then incubated with primary antibody overnight at 4 °C. The sections were treated with a biotinylated secondary antibody for 1 h at 37 °C. DAB Substrate (Hoffmann-La Roche Ltd., Basel, Switzerland) was then applied on the sections for 30 min. Finally, sections were washed with PBS and then incubated in hematoxylin for 15 min.

### 4.11. Statistical Analysis

All experimental data are expressed as the mean ± SD. Statistical evaluation was carried out using one-way analysis of variance (one-way ANOVA) followed by Scheffe’s multiple range test. *p* values < 0.05 were considered to indicate a significant difference.

## 5. Conclusions

In conclusion, the present study provides detailed insights into the molecular and cellular mechanisms of Dox-induced cardiotoxicity. Our results demonstrate that anthocyanin reversed Dox-induced cardiomyopathy via ERα/β. We show that Dox increases IGF-IIR expression to trigger apoptosis via decreasing CHIP to destabilize HSF1 in vitro and in vivo. Administration of anthocyanin activates ERα/β to reduce Dox-induced oxidative stresses, thereby stabilizing CHIP-mediated HSF1 nuclear translocation and SIRT1-mediated HSF1 activation to inhibit IGF-IIR expression. Taken together, our findings provide a novel strategy for preventing the Dox-induced cardiotoxicity.

## Figures and Tables

**Table 1:** Echocardiographic assessments of cardiovascular functions.

Cardiac Profiles	Control Group (*n* = 3)	Dox Group (*n* = 3)	Dox-CAN Group (*n* = 3)
LVIDd (mm)	8.09 ± 0.08	7.15 ± 0.15 **	8.12 ± 0.22 ###
LVIDs (mm)	5.23 ± 0.57	4.11 ± 0.14 **	4.59 ± 0.35
LVPWd (mm)	1.33 ± 0.07	0.96 ± 0.07 **	1.2 ± 0.07 #
LVPWs (mm)	2.18 ± 0.15	1.58 ± 0.15 *	2.23 ± 0.23 #
IVSd (mm)	1.16 ± 0.01	0.97 ± 0.07 *	1.21 ± 0.09 ##
IVSs (mm)	2.34 ± 0.08	1.69 ± 0.14 **	2.47 ± 0.26 ##
EDV (Teich)	1.28 ± 0.38	0.82 ± 0.05	1.19 ± 0.11
ESV (Teich)	0.39 ± 0.11	1.23 ± 0.02	1.24 ± 0.06
EF (Teich)	75.28 ± 2.29	62.32 ± 4.86 **	78.18 ± 1.72 ##
%FS	39 ± 2.57	31.38 ± 2.64 **	41.7 ± 1.47 ##
LVd Mass (ASE)	1.15 ± 0.06	0.96 ± 0.06	1.14 ± 0.1
LVs Mass (ASE)	1.21 ± 0.09	1.02 ± 0.03 *	1.2 ± 0.08 #

Values are mean ± S.D., * *p* < 0.05 and ** *p* < 0.01 (compared to control group). # *p* < 0.05, ## *p* < 0.01 and ### *p* < 0.001 (compared to Dox group). IVSd, interventricular septum at diastole; LVIDd, left ventricular diameter in diastole; LVPWd, left ventricular posterior wall thickness at diastole; IVSs, interventricular septal thickness in systole; LVIDs, left ventricular diameter in systole; LVPW, left ventricular posterior wall thickness; EDV, end-diastolic volume; ESV, end-systolic volume; EF, ejection fraction; FS, fractional shortening; LVd and LVs mass, left ventricular mass during diastole and systole.
